# Supplementary figures and images for: Endoscopic In Vivo Hyperspectral Imaging for Head and Neck Tumor Surgeries Using a Medically Approved CE-Certified Camera with Rapid Visualization During Surgery
Source: Cancers (Basel). 2024 Nov 10;16(22):3785. doi: 10.3390/cancers16223785 (PMC11592278; doi:10.3390/cancers16223785)

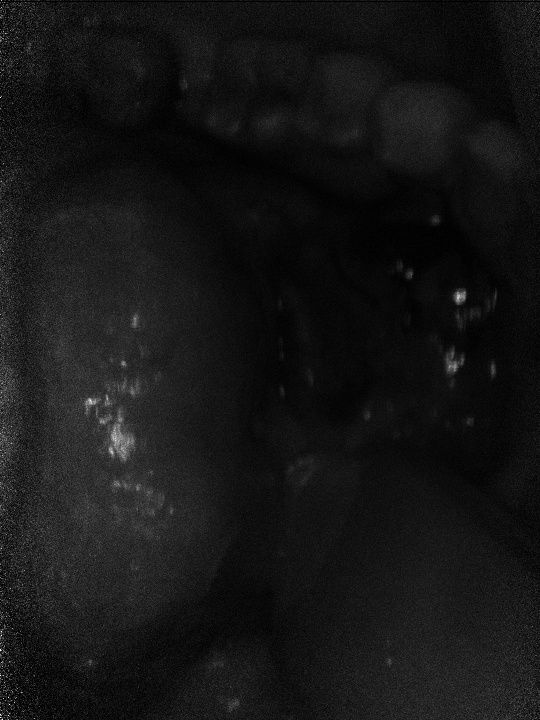

Supplement: Supplementary file 1 [file cancers-16-03785-s001.zip › S1/hyperspectral_multichannel.tif]

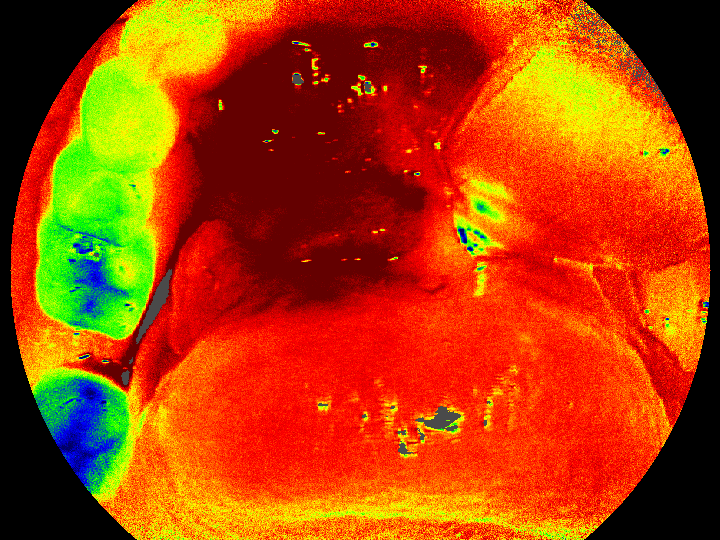

Supplement: Supplementary file 1 [file cancers-16-03785-s001.zip › S1/NIR-PI [(655-735), (825-925)nm].png]

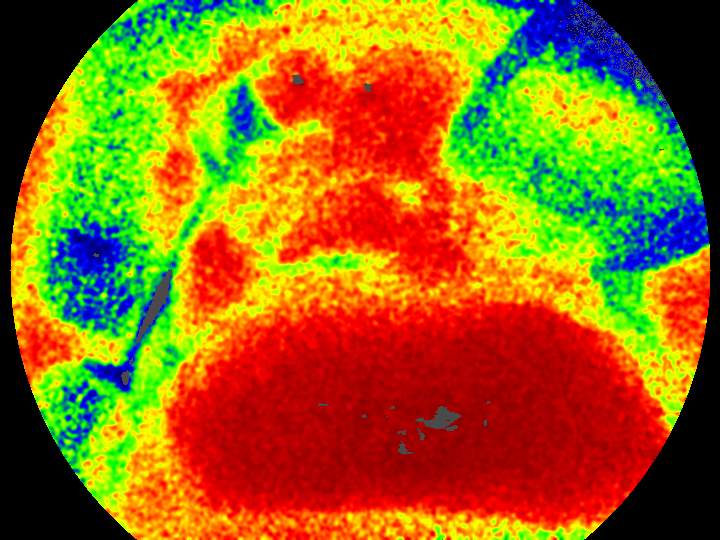

Supplement: Supplementary file 1 [file cancers-16-03785-s001.zip › S1/StO2 [(575-585), (740-780)nm].png]

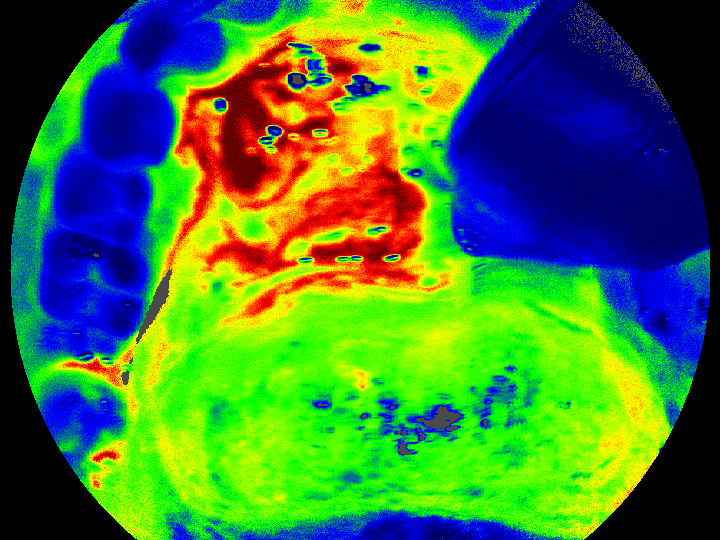

Supplement: Supplementary file 1 [file cancers-16-03785-s001.zip › S1/THI [(530-590), (785-825)nm].png]
